# Effect of drying on the bioactive compounds, antioxidant, antibacterial and antityrosinase activities of pomegranate peel

**DOI:** 10.1186/s12906-016-1132-y

**Published:** 2016-05-26

**Authors:** Rebogile R. Mphahlele, Olaniyi A. Fawole, Nokwanda P. Makunga, Umezuruike L. Opara

**Affiliations:** Postharvest Technology Research Laboratory, South African Research Chair in Postharvest Technology, Department of Horticultural Sciences, Faculty of AgriSciences, Stellenbosch University, Private Bag X1, Stellenbosch, 7602 South Africa; Postharvest Technology Research Laboratory, South African Research Chair in Postharvest Technology, Department of Food Science, Faculty of AgriSciences, Stellenbosch University, Private Bag X1, Stellenbosch, 7602 South Africa; Department of Botany and Zoology, Faculty of Science, Stellenbosch University, Private Bag X1, Stellenbosch, 7602 South Africa

**Keywords:** Freeze drying, Oven drying, Rutin, Total phenolics, Vitamin C

## Abstract

**Background:**

The use of pomegranate peel is highly associated with its rich phenolic concentration. Series of drying methods are recommended since bioactive compounds are highly sensitive to thermal degradation. The study was conducted to evaluate the effects of drying on the bioactive compounds, antioxidant as well as antibacterial and antityrosinase activities of pomegranate peel.

**Methods:**

Dried pomegranate peels with the initial moisture content of 70.30 % wet basis were prepared by freeze and oven drying at 40, 50 and 60 °C. Difference in CIE-LAB, chroma (C*) and hue angle (h°) were determined using colorimeter. Individual polyphenol retention was determined using LC-MS and LC-MS^E^ while total phenolics concentration (TPC), total flavonoid concentration (TFC), total tannins concentration (TTC) and vitamin C concentration were measured using colorimetric methods. The antioxidant activity was measured by radical scavenging activity (RSA) and ferric reducing antioxidant power (FRAP). Furthermore, the antibacterial activity of methanolic peel extracts were tested on Gram negative (*Escherichia coli* and *Klebsiella pneumonia*) and Gram positive bacteria (*Staphylococcus aureus* and *Bacillus subtilis*) using the in vitro microdilution assays. Tyrosinase enzyme inhibition was investigated against monophenolase (tyrosine) and diphenolase (DOPA), with arbutin as positive controls.

**Results:**

Oven drying at 60 °C resulted in high punicalin concentration (888.04 ± 141.03 mg CE/kg dried matter) along with poor red coloration (high hue angle). Freeze dried peel contained higher catechin concentration (674.51 mg/kg drying matter) + catechin and –epicatechin (70.56 mg/kg drying matter) compared to oven dried peel. Furthermore, freeze dried peel had the highest total phenolic, tannin and flavonoid concentrations compared to oven dried peel over the temperature range studied. High concentration of vitamin C (31.19 μg AAE/g dried matter) was observed in the oven dried (40 °C) pomegranate peel. Drying at 50 °C showed the highest inhibitory activity with the MIC values of 0.10 mg/ml against Gram positive (*Staphylococcus aureus* and *Bacillus subtili*. Likewise, the extracts dried at 50 °C showed potent inhibitory activity concentration (22.95 mg/ml) against monophenolase. Principal component analysis showed that the peel colour characteristics and bioactive compounds isolated the investigated drying method.

**Conclusions:**

The freeze and oven dried peel extracts exhibited a significant antibacterial and antioxidant activities. The freeze drying method had higher total phenolic, tannin and flavonoid concentration therefore can be explored as a feasible method for processing pomegranate peel to ensure retention of the maximum amount of their naturally occurring bioactive compounds.

**Trial registration:**

Not relevant for this study.

## Background

Pomegranate (*Punica granatum* L.) fruit is an important commercial crop cultivated in different parts of the world. The adaptability and health benefits are some of the characteristics responsible for its wide scale cultivation. About 50 % of the total fruit weight corresponds to the peel, which is an important source of bioactive compounds [1]. Meanwhile the edible part of pomegranate fruit consists of 40 % arils and 10 % seeds [2]. Pomegranate peel is a waste from juice processing. Several studies have confirmed that pomegranate peel is a rich source of bioactive compounds including ellagitannins, catechin, rutin and epicatechin among others [1, 3–5]. These bioactive compounds possess different biological activities such as scavenging reactive oxygen species (ROS), inhibiting oxidation and microbial growth and reducing the risk of chronic disease such as cancers and cardiovascular disorders [1, 4, 6].

However, the concentrations of bioactive compounds widely fluctuate among cultivars, environmental conditions, fruit maturity status, storage and postharvest treatments which may affect fruit quality and health beneficial compounds [7–11]. In the past, pomegranates was commonly used in conventional medicine for eliminating parasites and vermifuge, and to treat and cure apthae, ulcers, diarrhoea, acidosis, dysentery, haemorrhage, microbial infections and respiratory pathologies [6]. According to Gil et al. [12], pomegranate peel has the higher antioxidant activity than the pith and juice.

Drying is an ancient process used to preserve and prolong shelflife of various food products [13]. The main aim of drying food products is to remove water in the solid to a level at which microbial spoilage and deterioration resulting from chemical reactions is significantly reduced [14–17]. This enables the product to be stored for longer periods since the activity of microorganisms and enzymes is inhibited through drying [18, 19]. Generally, drying involves the application of thermal energy which cause water to evaporate into the vapour phase. However, drying results in structural, chemical and phytochemical changes that can affect quality properties such as texture, colour and nutritional values [20–22]. Several drying techniques used for various products include air, oven and freeze drying. Generally, air-drying and oven drying are favoured due to processing cost and efficiency [23]. However, air drying has drawbacks of both long drying time required and poor quality [24, 25]. By far, freeze drying is regarded as the better method for moisture removal, with final products of the highest quality compared with air-drying [13, 26].

Pomegranate ‘Wonderful’ is the most widely grown and consumed pomegranate cultivar globally [27] and during the past 10 years, South Africa has seen tremendous increase in commercial production of the registered cultivar, accounting for over 1000 ha of total planted area and 56 % of total production [28]. Pomegranate peel has been known for many years for its health benefit, including antibacterial activity. More recently, research indicated that pomegranate peel extracts also inhibit tyrosinase activity [4] an enzyme that induces the production of melanin which leads to hyperpigmentation of the skin.

The high level of bioactive compounds in the peel as well as the reported health benefits to date make these desirable by-products as functional ingredients in food, nutraceuticals and pharmaceutics [4, 5, 29]. Previous researches have been limited to the characterization of phenolic compounds of the pomegranate peel extracts and the evaluation of its biological activities. However, the information on the effect of drying on the pharmacological properties is limited. Therefore, the aim of this study was to investigate the concentrations of polyphenols compounds, antioxidant activity and the in vitro pharmacological properties of pomegranate peel using freeze and oven drying (at various temperature range).

## Methods

### Fruit source

Pomegranate ‘Wonderful’ fruit (a commercial registered cultivar in South Africa) were sourced during commercial harvest from Sonlia packhouse (33°34′851″S, 19°00′360″E) in Western Cape, South Africa. The ‘Wonderful’ is the only late cultivar grown in South Africa, harvested between April and May of every year. The fruit was further verified by Mr. Neil Maree of the Pomegranate Association of South African (POMASA) and a voucher specimen retained with as PUC: W1153. Fruit were transported to the Postharvest Technology Laboratory at Stellenbosch University. Fruit of the same size shape, colour and without any physical defects were randomly selected. Fresh pomegranate peel was cut in the dimension of 20 ± 0.5 mm (length), 20 ± 0.5 mm (width) and 5 ± 0.5 mm (thickness) were used. Before drying, the peels were stored at −80 °C until use. Moisture content was measured using a modified AOAC method 925.45 [30] with slight modifications by drying the peel using the oven at 105 ± 0.5 °C for 24 h. The oven was kept functional for an hour to equilibrate the oven temperature before drying. The accuracy of the oven temperature was monitored using thermometer (Thermco®, Germany). All the drying tests were run two times in triplicates at each temperature and averages were reported.

### Drying procedure

#### Oven drying

Three different temperature levels (40, 50 and 60 °C) were used and the oven dryer (Model nr. 072160, Prolab Instruments, Sep Sci., South Africa) was operated at an air velocity of 1.0 m/s, parallel to the drying surface of the sample. Weight change was recorded by a digital balance (ML3002.E, Mettler Toledo, Switzerland) at an hourly interval during drying. Peels were dried until equilibrium (no weight change) was reached.

#### Freeze drying

Prior to drying, peels were frozen at −80 °C for 2 days. Frozen pomegranate peels were freeze dried using a freeze dryer (VirTis Co., Gardiner, NY, USA) at a vacuum pressure of 7 mTorr and the condenser temperature of −88.7 °C. Similar procedure for monitoring weight loss as explained above was employed. Weight loss was recorded at 2 h intervals. The drying time needed to reach equilibrium weight and the residual moisture content in all drying methods is presented in Table 1.

**Table 1:** Residual moisture using freeze and oven drying methods

Drying method	Drying time (h)	Residual moisture (kg water/kg dry matter)
Freeze dried	16	0.087 ± 0.002^b^
40 °C	22	0.093 ± 0.002^a^
50 °C	17	0.094 ± 0.002^a^
60 °C	12	0.096 ± 0.004^a^

Means in the same column with different letter(s) ^(a-b)^ differ significantly (*P* < 0.05) according to Duncan’s multiple range tests

### Colour

Peel colour change was measured before and after drying using the CIE L*, a*, b* coordinates with a calibrated Minolta Chroma Meter (Model CR-400/410, Minolta Corp, Osaka, Japan). The hue angle (h°) and colour intensity (C*) were calculated [31]. The values provided for each sample were the average of three replicates. The total colour difference (*∆*E*) were calculated using the following formula:

1 $$ \varDelta {E}^{*}={\left[{\left(\varDelta {L}^{*}\right)}^2+{\left(\varDelta {a}^{*}\right)}^2+{\left(\varDelta {b}^{*}\right)}^2\right]}^{1/2} $$

where ∆L*, ∆a* and ∆b* are the differences between the colour of the fresh and dried sample.

### Peel preparation

Dried peel was ground to a fine powder using a miller (Model A11, IKA, Germany) and screened through a plastic mesh sieve, with a mesh size of 1.4 mm particle size (Vanguard, India). Dried pomegranate peel (2 g) from each drying methods were extracted separately with 10 ml of 80 % (*w/v*) methanol using sonication for approximately 1 h [32]. The extracts were separately filtered with Whatman no.1 filter paper and residues were re-extracted following the same procedure. The extracts were pooled before drying under stream of air.

### Determination of individual phenolic acids and flavonoids concentration

LC-MS and LC-MS^E^ analyses were conducted on a Waters Synapt G2 quadrupole time-of-flight mass spectrometer system (Milford, MA, USA). The instrument was connected to a Waters Acquity ultra-performance liquid chromatograph (UPLC) and Acquity photo diode array (PDA) detector. Ionisation was achieved with an electrospray source using a cone voltage of 15 V and capillary voltage of 2.5 kV using negative mode for analysis of phenolic compounds. Nitrogen was used as the desolvation gas, at a flow rate of 650 L/h and desolvation temperature of 275 °C. The separations were carried on a waters UPLC BEH C18 column (2.1 × 50 mm, 1.7 μm particle size), with injection volume of 3 μl at flow rate of 0.4 ml/min. The gradient for the analysis of phenolic compounds started with 100 % using 0.1 % (*v/v*) formic acid (solvent A) and kept at 100 % for 0.5 min, followed by a linear gradient to 22 % acetonitrile (solvent B) over 2.5 min, 44 % solvent B over 4 min and finally to 100 % solvent B over 5 min. The column was subjected to 100 % solvent B for an extra 2 min. The column was then re-equilibrated over 1 min to yield a total run time of 15 min. Reference standards (Sigma-Aldrich, South Africa) of phenolic acids and flavonoids were used for the quantification of individual compounds in pomegranate peel extracts.

### Determination of total phenolic concentration

Total phenolic concentration (TPC) was measured using the Folin-Ciocalteu (Folin-C) method as described by [33] with slight modification [4]. Diluted peel extracts (50 μl) was mixed with 450 μl of 50 % methanol followed by the addition of 500 μl Folin-C and then sodium carbonate (2 %) solution after 2 min. The mixture was vortexed and absorbance read at 725 nm using a UV-visible spectrophotometer (Thermo Scientific Technologies, Madison, Wisconsin). Gallic acid standard curve (0.08− 0.32 mg/ml) was used and TPC was expressed as milligram gallic acid equivalent per kilogram peel extracts (mg GAE/kg DM).

### Determination of total tannin concentration

Total tannin analysis was carried out using Folin-C method described by [33]. Polyvinylpolypyrrolidone (PVPP) was used to separate tannin from non-tannin compound in peel extracts by adding 100 mg of PVPP to 1.0 ml of distilled water and 1.0 ml peel extracts in a test tube. The mixture was vortexed and kept at 4 °C for 15 min followed by centrifugation at 4000 g for 10 min. After the extraction, 50 μl of supernatant was mixed with 450 μl of 50 % methanol followed by the addition of 500 μl Folin-C and then sodium carbonate (2 %) solution after 2 min. The absorbance was recorded at 725 nm using UV-Visible spectrophotometer after incubation for 40 min at room temperature. Separate peel extracts not treated with PVPP was measured for total phenolic concentration. Total tannin concentration was calculated as:

2 $$ \mathrm{Total}\kern0.5em \mathrm{tannin}\kern0.5em \mathrm{concentrations}\kern0.5em \left(\mathrm{T}\mathrm{T}\mathrm{C}\right)={\mathrm{TPC}}_{\left(\mathrm{in}\kern0.5em \mathrm{peel}\kern0.5em \mathrm{extract}\kern0.5em \mathrm{with}\mathrm{out}\kern0.5em \mathrm{PVPP}\right)}\hbox{--} {\mathrm{TPC}}_{\left(\mathrm{in}\kern0.5em \mathrm{peel}\kern0.5em \mathrm{extract}\kern0.5em \mathrm{treated}\kern0.5em \mathrm{with}\kern0.5em \mathrm{PVPP}\right)} $$

where TPC referred to total phenolic concentration (mg GAE/kg DM).

Results were expressed as milligram gallic acid equivalent per kilogram peel extracts (mg GAE/kg DM).

### Determination of total flavonoid concentration

Total flavonoid concentration was measured spectrophotometrically as described by Yang et al. [34]. PJ (1.0 g) was extracted with 50 % methanol (49 ml) and vortexed for 30 s. The mixture was sonicated in an ultrasonic bath for 10 min and centrifuged at 4000 g for 12 min at 4 °C. Distilled water (1.2 ml) was added to 250 μl of extracted peel extracts and then followed by 75 μl of 5 % sodium nitrite. After 5 min, freshly prepared 10 % aluminium chloride (150 μl) was added to the mixture, followed by the addition of 500 μl sodium hydroxide after a another 5 min, and 775 μl distilled water bringing the final volume to 3 ml. The mixture was vortexed and absorbance was immediately read using spectrophotometer at 510 nm. Catechin (0.01–0.5 mg/ml) was used for the standard curve. The results were expressed as catechin equivalent per kilograms peel extracts (mg CE/kg DM).

### Radical scavenging activity (RSA)

The ability of peel extract to scavenge 2, 2-diphenyl-1-picryl hydrazyl (DPPH) radical was measured following the procedure described by Karioti et al. [35] with slight modifications [4]. Peel extract (15 μl) was mixed with 735 μl methanol and 0.1 mM solution of DPPH (750 μl) dissolved in methanol. The mixture was incubated for 30 min in the dark at room temperature before measuring the absorbance at 517 nm using a UV-visible spectrophotometer (Thermo Scientific Technologies, Madison, Wisconsin). The RSA was determined by ascorbic acid standard curve (0–1500 μM). The results were presented as millimolar ascorbic acid (AA) equivalent per gram of peel extracts (mM AAE/g DM).

### Ferric reducing antioxidant power

Ferric reducing antioxidant power assay was performed according to the method of Benzie and Strain [36]. FRAP solutions contained 25 ml acetate buffer (300 mM acetate buffer, pH 3.6), 2.5 ml (10 mM of TPTZ solution), 2.5 ml (20 mM of FeCl_3_ solution). Ten millilitre of aqueous methanol (50 %) was added to peel extract (1 ml), sonicated for 10 min in cold water and centrifuged for 5 min at 4 °C. PJ (150 μl) was mixed with 2850 μl FRAP and the absorbance was read at 593 nm after 30 min incubation using a UV-visible spectrophotometer. Trolox (0–1.5 mM) was used for calibration curve, and results were expressed as trolox (μM) equivalents per millilitre pomegranate juice (μM TE/g DM).

### Determination of ascorbic acid concentration

Ascorbic acid was determined according to Klein and Perry [37] with slight modifications [38]. Briefly, peel extract (1.0 g) was mixed with 50 ml of 1 % metaphosphoric acid followed by sonication on ice for 4 min and centrifugation at 4000 g for 12 min. Supernatant (1.0 ml) was pipetted into a tube and mixed with 9 ml of 2, 6 dichlorophenolindophenol dye (0.0025 %). The mixture was incubated in the dark for 10 min before absorbance was measured at 515 nm. Calibration curve of authentic L-ascorbic acid (0.01–0.1 μg/ml) was used to calculate ascorbic acid concentration. Results were expressed as ascorbic acid equivalents per millilitre crude juice (μg AAE/g DM).

### Antibacterial assay

#### Microdilution assay

Antibacterial activity of pomegranate peel was determined following microdilution assay for the minimum inhibitory concentration values [4]. Four bacterial strains used comprised two Gram-negative bacteria (*Escherichia coli* ATCC 11775 and *Klebsiella pneumonia* ATCC 13883) and two Gram-positive bacteria (*Bacillus subtilis* ATCC 6051 and *Staphylococcus aureus* ATCC 12600). All the bacteria were grown in sterile MH broth. The stock solutions of the peel extracts were dissolved in methanol to make 50.0 mg/ml. Under aseptic conditions, 100 μl of sterile water were added in a 96-well micro plate followed by 100 μl peel extracts as well as bacterial culture and serially diluted (two-fold). Similarly, two fold serial dilution of streptomycin (0.1 mg/ml) was used as positive control against each bacterium. Bacteria-free broth, methanol solvent (100 %) and sterile water were included as negative controls. The final concentration of peel extract ranged from 0.097 to 12.5 mg/ml, whereas streptomycin was between (0.097–12.5 mg/ml). Plates were incubated for 18 h at 37 °C. After incubation, bacterial growth in the plate was indicated by adding 40 μl of *p*-iodonitrotetrazolium chloride (Sigma-Aldrich, Germany) after incubation. Bacterial growth was indicated by pink colour, while clear wells indicated inhibition. The results were recorded in terms of the minimal inhibitory concentration which is regarded as the lowest concentration of the extract without bacterial growth. The assay was measured in triplicates.

#### Mushroom tyrosinase inhibition assay

Tyrosinase inhibitory activity was determined using colorimetric method as described by Momtaz et al. [39] with slight modification [4]. _L_-tyrosine and L-3,4-dihydroxyphenylalanine (_L_-DOPA, Sigma) were used as substrates. Assays were carried out in a 96-well micro-titre plate and a Multiskan FC plate reader (Thermo scientific technologies, China) was used. Peel extracts and seed oil were dissolved in methanol and DMSO, respect to concentration of 50 mg/ml and further diluted in potassium phosphate buffer (50 mM, pH 6.5) to 1000 ug/ml. Each prepared sample (70 μl) was mixed with 30 μl of tyrosinase (333 Units/ml in phosphate buffer, pH 6.5). After 5 min incubation, 110 μl of substrate (2 mM _L_-tyrosine or 12 mM _L_-DOPA) was added to the reaction mixtures and incubated for 30 min. The final concentration of the extracts were between 2.6 and 333.3 μg/ml. Arbutin (1.04–133.33 μg/ml) was used as a positive control while a blank test was used as each sample that had all the components except _L_-tyrosine or _L_-DOPA. The final concentrations of the seed oil were between 017 and 5 mg/ml whereas the positive control (arbutin) were between 4.10 and 400 μg/L. All the steps in the assay were conducted at room temperature. Results were compared with a control consisting of DMSO instead of the test sample. After adding mushroom tyrosinase solution, the reaction mixture was incubated at room temperature (37 °C) for 30 min. The absorbance of the reaction mixture was measured at 475 nm. The percentage mushroom tyrosinase inhibitory activity was calculated using the following equation:

$$ \%\kern0.5em \mathrm{inhibition}=\left[\left({\mathrm{A}}_{\mathrm{control}}-\mathrm{Asample}\right)/{\mathrm{A}}_{\mathrm{control}}\right]\times 100 $$

where A_control_ is the absorbance of Methanol and A_extract_ is the absorbance of the test reaction mixture containing extract or arbutin. The EC_50_ values of extracts and arbutin were calculated. The assay was measured in triplicate.

### Statistical analysis

Statistical analyses were carried out using statistical software (STATISTICA, Vers. 12.0, StatSoft Inc., USA). Data was subjected to analysis of variance (ANOVA) and means were separated by least significant difference (LSD; *P* = 0.05) according to Duncan’s multiple range test. GraphPad Prism software version 4.03 (GraphPad Software, Inc., San Diego, USA) was used for graphical presentations. Principal component analysis (PCA) was carried out using XLSTAT software version 2012.04.1 (Addinsoft, France). Triplicate measurements were carried out and the values are reported as mean ± standard error.

## Results and discussion

### Drying time and residual moisture content

The drying time required to achieve a final moisture concentration of the peel were, 22, 17, 16 and 12 h for 40, 50 °C, freeze drier and 60 °C, respectively (Table 1). Drying time resulted in the residual moisture concentration which varied from 0.087 to 0.096 kg water/kg dry matter. Overall, freeze dried peel had the lowest residual moisture whereas oven dried peels did not vary in all temperature range.

### Peel colour

The freeze dried peel showed a considerably higher L* (lightness/brightness) with no significant differences in colour lightness of peel dried in the oven at 40, 50 and 60 °C, indicating a darker coloration than that of freeze dried peel (Table 2). High decline in lightness (L*) was observed by Toor and Savage [40] and Ashebir et al. [41] in different tomatoes cultivars dried at various temperature. Moreover, a* (peel redness) value is in the order of 60 °C > 50 °C > 40 °C > freeze dried. The chroma value indicates the degree of saturation of colour and is proportional to the strength of the colour [42]. In this study, the freeze dried peel had the lowest colour intensity compared to oven dried peel at the investigated temperatures. The results indicate that freeze dried peel were slightly bleached (lower chroma value) which was also confirmed by lower a* value compared to the oven dried peel. Moreover, there was a negligible increase in hue angle (darkness) and followed the order: 60 °C > 40 °C > 50 °C > freeze dried. According to Bahloul et al. [43] the increase in hue angle is indicative of browning reaction as a result of activity of polyphenolic oxidase. Likewise, formation of brown compounds may be as a result of Maillard reaction which occurs upon the reduction of sugar and amino acids [44]. Comparable results were also reported by other authors. For instance, Wojdylo et al. [45] reported high L* (lightness) but less a* (red colour) value in freeze dried sour cherries. However, Vega-Galvez et al. [46] reported an increased L* and a* value in pre-treated red bell pepper dried at an air temperature in the range of 50 and 80 °C. The change in total colour difference (∆E) is an important part for dried product, which expresses human eye’s ability to differentiate between colours of various samples [45]. Slight but notable variation was observed in the total colour difference between the drying methods, with oven drying at 60 °C having the lowest TCD (16.82) while the highest was observed in peel dried at 50 °C (23.10) (Table 2).

**Table 2:** Pomegranate peel colour attributes after drying

Drying method	Colour attributes				
	L*	a*	C*	*h*°	Δ*E*
Fresh peel	51.01 ± 1.67^b^	28.85 ± 1.70^a^	35.14 ± 1.60^a^	34.61 ± 2.05^b^	–
Freeze dried	61.46 ± 1.59^a^	23.33 ± 1.28^b^	29.99 ± 0.86^b^	38.85 ± 2.18^ab^	17.77 ± 1.07^b^
40 °C	41.51 ± 1.09^c^	24.33 ± 1.01^b^	33.13 ± 0.77^a^	42.32 ± 1.70^a^	18.72 ± 1.14^ab^
50 °C	39.29 ± 1.37^c^	25.24 ± 0.88^b^	33.25 ± 0.78^a^	39.82 ± 1.76^ab^	23.10 ± 2.42^a^
60 °C	42.04 ± 0.92^c^	25.24 ± 1.16^b^	35.08 ± 0.66^a^	43.78 ± 1.90^a^	16.82 ± 2.00^b^

Means in the same column with different letter(s) ^(a-b)^ differ significantly (*P* < 0.05) according to Duncan’s multiple range tests. L* = lightness/darkness; a* = redness/greenness; C* = chroma; h° = hue angle; ΔE = total colour difference

### Individual phenolic acid and flavonoid compound

The phenols identified in dried pomegranate peel include phenolic acid (p-coumaric), flavan-3-ols (+catechin, -epicatechin), flavanone (hesperidin), flavonol (rutin), ellagitannin (punicalin) (Table 3). Punicalin is hydrolysable tannin which is known to account for high antioxidant activity in pomegranate peel [47, 48]. Punicalin values ranged from 559.60 to 888.40 mg/kg DM. As can be observed, drying at 60 °C resulted in relatively higher punicalin concentration, which was 32.98, 25.41, 15.66 % higher than oven dried (40 °C), (50 °C) and freeze dried peel, respectively. Higher retention of punicalin compound at 60 °C may be as a result of less exposure to oxygen as the drying time was shorter (12 h). The highest concentration of rutin was found in freeze dried peel (4666.03 mg/kg DM) followed by drying at 60 °C (3401.36 mg/kg DM) and 40 °C (2135.00 mg/kg DM) whereas it was not detected in the peel dried at 50 °C. It could be suggested that rutin could have undergone enzymatic degradation/copigmentation reactions with other molecules since flavonols are among the most effective co-pigments [49]. On the other hand, p-coumaric was only detected in the oven dried peel at 50 and 60 °C. However, p-coumaric was not detected in the peel dried using freeze and oven drying (40 °C). This could be due to the fact that prolonged exposure of pomegranate peel to drying treatment (40 °C) may result in the formation of new compounds (Maillard products). Likewise, the degradation of the hydroxycinnamic acids during drying at 40 °C could have been influenced by polyphenoloxidase (PPO) enzymatic activity. It has been observed that during the dehydration process PPO activity remains high for long periods when the drying temperature are around 55 °C whereas only moderate activity is observed at temperatures higher than 75 °C [50]. Devic et al. [51] reported that hydroxycinnamic acids are poorly preserved by the drying process. For instance, hydroxycinnamic acids could firstly be involved in enzymatic browning but can also diffuse more easily as their molecular weight is lower. In relation to freeze drying method, enzymatic oxidation by polyphenol oxidase and peroxidase might occur as result of lower exposure to oxygen [52] and cell structure injury caused by ice crystals formation leading to the exposure of phenolics to oxidative conditions [53]. Generally, it was observed that the concentrations of rutin, +catechin, -epicatechin, and hesperidin were significantly higher in freeze dried compared to oven dried for the whole temperature range (40, 50 and 60 °C). It has been highlighted that high porosity of dehydrated food promotes greater contact of the material with oxygen and facilitate oxidation of compounds [54], whereas drying treatments release bound phytochemicals from the matrix to make them more accessible in extraction [45]. Comparable results were reported by [55], who observed high phenolic concentration of freeze dried mulberry leaves. Freeze dried peel induced the increase bioactive compounds than oven drying as observed in this study.

**Table 3:** Individual phenolic and flavonoid concentration in fresh and dried pomegranate peel

Drying method						
	Punicalin (mg CE/kg DM)	Rutin	p-Coumaric	+Catechin (mg/kg DM)	-Epicatechin	Hesperidin
Freeze dried	708.38 ± 48.86^b^	4666.03 ± 311.70^a^	nd	674.51 ± 21.30^a^	70.56 ± 0.22^a^	16.45 ± 1.65^a^
40 °C	768.11 ± 1.67^b^	2135.00 ± 0.00^c^	nd	377.26 ± 22.05^c^	28.93 ± 1.55^c^	5.07 ± 0.02^b^
50 °C	672.98 ± 26.93^b^	nd	0.45 ± 0.02	340.64 ± 21.06^c^	31.95 ± 3.37^bc^	4.59 ± 0.54^b^
60 °C	888.04 ± 57.57^a^	3401.36 ± 0.00^b^	0.57 ± 0.52	443.41 ± 0.30^b^	34.74 ± 0.11^b^	1.77 ± 0.54^c^

Mean in column with different letter(s) ^(a-c)^ differ significantly (*P* < 0.05) according to Duncan’s multiple range test. *nd* not detected. (*n* = 3). Means ± SE presented

### Total phenolic (TPC), total tannins (TTC) and flavonoid concentration (TFC)

**Fig. 1:**
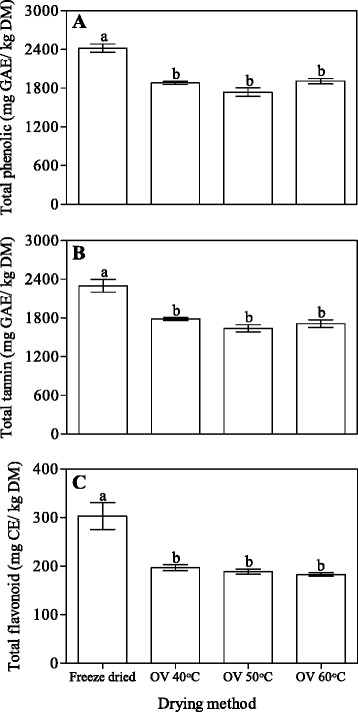
Effects of drying methods on concentrations of total phenolic (**a**), total tannins (**b**) and total flavonoid (**c**) of pomegranate peel. *Bars* with *same letter* are not significantly different (*P* < 0.05; Duncan’s multiple range test). Data represent the Mean ± SE (*n* = 3)

Concentrations of total phenolic, total tannin and flavonoid after drying in pomegranate peel are shown in Fig. 1. On the dry basis, TPC, TTC and TFC were between 3 and 5 folds more in freeze dried peel than the oven dried peel at all temperatures. The results were consistence with those reported by [56] who observed the highest total phenolic in freeze dried persimmon powder because of limited thermal and chemical degradation, as it was performed at low temperatures. Asami et al. [57] reported that hot-air drying promoted the oxidation and condensation of phenolic compounds compared to freeze-drying. Similar results were reported by Calín-Sánchez et al. [58] who reported higher total phenolic concentration in pomegranate rind after freeze drying. Generally, thermal treatment has significant effect on the depletion of polyphenols in food products [59]. Vega-Gálvez et al. [23] also reported loss of polyphenol compounds in air dried red pepper in which polyphenol concentration decreased with drying temperature. In this study, total phenolic concentration did not vary across all the temperatures for oven dried method. This may be due to the fact that some phenolic compounds are destroyed by the elevated temperature during the drying process. Our results were similar to the results of Wolfe and Liu [60] who did not observe any significant difference in total phenolics and flavonoids in apple peels dried under oven conditions (40, 60, or 80 °C). According to Wojdyło et al. [45] this behaviour may be due to the fact that a large percentage of phenolic compounds are bound to cellular structures, and dehydration treatments release bound phytochemicals from the matrix to make them more accessible in extraction. Freeze drying is often considered to be the most effective technique for preserving temperature sensitive compound since the ice crystals formed within the plant matrix can rupture the cell structure, which provides the release of cellular components [61]. With regard to the present study, freeze drying would be a better drying method for preserving total phenolic, tannins and flavonoid concentration of pomegranate peel than oven drying process.

### Radical scavenging activity (RSA) and ferric reducing antioxidant power (FRAP) and vitamin C concentration

**Fig. 2:**
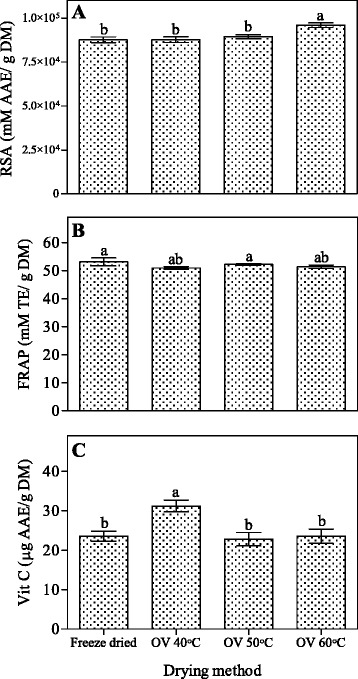
Effects of drying methods on RSA (**a**), FRAP (**b**) and vitamin C concentration (**c**) of pomegranate peel. *Bars* with *same letter* are not significantly different (*P* < 0.05; Duncan’s multiple range test). Data represent the Mean ± SE (*n* = 3). *RSA* radical scavenging activity, *FRAP* ferric reducing antioxidant power, *Vit C* Vitamin C

Peel dried at 60 °C significantly (*P* < 0.05) had higher radical scavenging activity compared to 40, 50 °C and freeze drier (Fig. 2a). As can be observed, increased radical scavenging activities in peel dried at 60 °C coincide with higher punicalin, +catechin, -epicatechin and rutin compound concentration. It has been reported that the antioxidant activity may be related with amount of compounds since they act as scavengers of free radicals produced during oxidation reaction [62]. Moreover, recent studies showed that the radical scavenging activity was elevated at higher drying temperatures using oven drying treatments [63, 64].

The reducing power of pomegranate peel was determined using ferric reducing antioxidant power method which measures reduction of Fe^+3^ to Fe^+2^. In this study, antioxidant activity (FRAP) of pomegranate peel did not vary significantly (*P* > 0.05) between the drying methods. The reducing power was in the order of 40 °C > 50 °C, 60 °C > freeze-dried (Fig. 2b). It can be observed that freeze and oven drying at various temperatures affected vitamin C (*P* < 0.05), thus a considerable higher vitamin C in samples dried at 40 °C was observed (Fig. 2c). However, no significant difference was observed between freeze and oven-dried (at 50 and 60 °C) peel. According to Marques and Freire [65], the small losses in vitamin C in freeze dried product are attributed to low temperature and to the use of vacuum in the process. Researchers observed loss of vitamin C during air-drying of pomegranate peel [1]. Our findings are in agreement with those of Vega-Gàlvez et al. [23] who reported loss of vitamin C during oven drying at temperatures between 50 and 90 °C in red pepper. Miranda et al. [66] reported the loss of 70 % of vitamin C after drying in *aloe vera* gel and the authors concluded that this may be the result of irreversible oxidation during drying with hot air. Therefore, lower vitamin C concentration observed in this study may be as a result of irreversible oxidation during drying. Vitamin C is a thermo-sensitive compound, therefore lower concentration was likely due to elevated processing temperature [67, 68] and period of exposure required to dry the sample at 50 and 60 °C.

### Antibacterial activity

The antibacterial activity of dried pomegranate peel extracts are presented in Table 4. As can be observed, all the extracts showed the broad-spectrum activity against the bacterial strains used. The minimum inhibitory activity values observed against the tested bacteria ranged from 0.10 to 0.39 mg/ml. Moreover, drying at 50 °C showed the highest inhibitory activity with the MIC values of 0.10 mg/ml against gram positive bacteria in particular *Staphylococcus aureus* and *Bacillus subtilis* compared with the rest of the treatments. Results from this study indicated that the peel extracts were more effective against the tested bacteria irrespective of the drying methods employed. Possible explanation could be as a result of higher retention of antioxidant activity after drying. Likewise, the activity against all the test bacteria (Gram-positive and Gram-negative bacteria) indicates that extracts contain broad spectrum metabolic toxins. Wojdylo et al. [45] indicated that polyphenols in an intermediates state of oxidation may exhibit higher radical scavenging efficiency than the non-oxidized ones although a subsequent loss in the antioxidant properties has been found for advanced enzymatic oxidation steps [61]. To some extent, this is consistent with previous studies on antibacterial activity pomegranate peel extracts [1, 4, 69]. It has been reported that the antibacterial activity of pomegranate peel extracts can be attributed to the presence of high molecular weight compounds such as tannins. In addition, the tannin rich ellagitannins have antibacterial and antifungal and antiprotozoal activity [70–72]. The results of the study showed that drying either by freeze or oven showed the best MIC which indicate high stability of compounds contained in the pomegranate peel.

**Table 4:** Antibacterial activity (MIC, mg/ml) of dried pomegranate peel extracts using two different drying methods

Treatment	Gram negative		Gram positive	
	*Escherichia coli*	*Klebsiella pneumonia*	*Staphylococcus aureus*	*Bacillus subtilis*
Freeze dried	0.39	0.39	0.20	0.20
40 °C	0.20	0.20	0.20	0.20
50 °C	0.20	0.20	0.10	0.10
60 °C	0.39	0.39	0.39	0.39
Streptomycin (mg/ml)	0.02	0.02	0.02	0.02

### Tyrosinase inhibitory activity

Tyrosinase plays a key role in biosynthesis of melanin which is responsible for pigments of the skin, eyes and hair in mammals as well as browning of the fruits [73]. There are two distinct reactions of melanin biosynthesis; the hydroxylation of _L_-tyrosine (monophenolase activity) and the conversion of _L_-DOPA (diphenolase activity) to the corresponding monophenolase and diphenolase are the key susbtrate facilitating the O-quinones [74]. These quinones are highly reactive, and tend to polymerize spontaneously to form brown pigments, namely melanin. The inhibitory effect of dried peel extracts on the inhibitory activity of tyrosinase is presented in Table 5. The inhibitory activity (IC_50_) against monophenolase was in the range of 22.95–107.73 mg/ml. The peel extract dried at 50 °C notably showed better inhibition on monophenolase activity. The highest inhibition activity against monophenolase was found to be 22.95 mg/ml of peel extracts concentration compared to the rest of treatment. Moreover, the extracts of peel dried at 50 °C showed potent inhibitory activity than the arbutin. In general, peel extracts showed weaker diphonalase inhibition in all treatments compared to arbutin (control). However, better inhibitory activity against diphenolase was observed in the peel extracts dried at 60 °C with MIC value of 62.09 mg/ml. Nevertheless, pomegranate peel contains a mixture of many kinds of secondary metabolism products, including phenolics, which vary greatly in their antioxidant capacity and phenolic compounds composition. The IC_50_ values reported in this study (21.41–112.79.34 mg/ml) were in the range with those observed by Fawole et al. [4] where IC_50_ value against menophenolase and diphenolase ranged from 3.66 to 25.56 and 15.88 to 114.9 μg/ml, respectively.

**Table 5:** Effective inhibition concentration (EC_50_) of fresh and dried fruit peel extracts against tyrosinase

Treatment	Monophenolase	Diphenolase
	(IC_50_ mg/ml)	
Freeze dried	107.73 ± 10.08^a^	86.93 ± 15.23^ab^
40 °C	45.07 ± 6.05^b^	119.79 ± 20.23^a^
50 °C	22.95 ± 1.53^c^	74.05 ± 10.27^ab^
60 °C	64.27 ± 10.35^b^	62.09 ± 2.98^b^
Arbutin (mg/ml)	44.00 ± 5.56^b^	14.99 ± 2.52^c^

Mean in column with different letter(s) ^(a-c)^ differ significantly (*P* < 0.05) according to Duncan’s multiple range test. IC_50_ (mg/ml), concentration for inhibitory 50 % of tyrosinase. Data represent the Mean ± SE (*n* = 3)

### Multivariate analysis

#### Principal component analysis

**Fig. 3:**
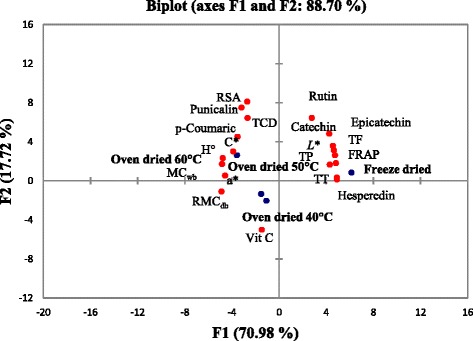
Principal component analysis of the first two factors (F1 and F2) based on colour attributes and bioactive compounds of pomegranate peel cv. Wonderful obtained from different drying methods. *TCD* total colour difference, *TF* total flavonoid, *TP* total phenolic, *TT* total tannin, *RSA* radical scavenging activity, *Vit C* Vitamin C, *FRAP* ferric reducing antioxidant power. L* lightness, C* chroma, *H°* hue angle, a* redness, *RMC* residual moisture content (dry basis %), *MC* moisture content (wet basis %)

The results show the average of individual phenolic, total phenolic, antioxidant activity and color coordinates of pomegranate dried peel by oven and freeze drying. The two principal components (PC1 and PC2) explain 88.70 % of the total data variance (Fig. 3). As observed, PC1 explained 70.98 % of the total variance whilst PC2 explained only 17.71 % of the total variability which showed that the disparity among pomegranate peel dried using different methods was described by the F1 (Fig. 3). The observations (Fig. 3) indicated that freeze dried peel could be associated with catechin, rutin, epicatechin hesperidin, total flavonoid, total phenolic, total tannin, ferric reducing antioxidant power and lightness which had higher positive scores along F1 (Table 6). Moreover, the higher negative scores (Table 6) along F1 (Fig. 3) correspond to chroma (C*), p-coumaric, radical scavenging activity, hue angle, punicalin and redness (a*), moisture content (wb %) and residual moisture content (db %) of the peel dried at 60 °C. Along F1 (Fig. 3), lower positive scores correspond to total colour difference, of oven dried peel at 50 °C. Likewise, high positive scores (Table 6) along F2 is associated with total colour difference, rutin, radical scavenging activity and punicalin of the oven dried (60 °C) (Fig. 3). Along F2, high negative scores (as shown in Fig. 3 and Table 6) for oven dried (40 °C) could characterize the peel for having high vitamin C concentration. However, lower positive scores along F2 were from freeze dried peel (associated with total phenolic concentration, total flavonoid concentration, ferric reducing antioxidant power, total tannin concentration, hesperidin, Chroma and hue angle). The lower negative scores (Fig. 3) along F2 (Table 6) were from oven dried (50 °C) (associated with residual moisture content). The results demonstrated that PCA showed that freeze drying and oven drying at all temperature range have significantly different properties.

**Table 6:** Factor loadings, eigenvalue, cumulative variance (%) and score for the first two principal (F1–F2) components based on pomegranate peel from two different drying methods

Loadings	F1	F2
Catechin	0.864	0.487
Epicatechin	0.930	0.363
Punicalin	−0.543	0.653
Hesperedin	0.999	0.033
RSA	−0.649	0.760
Vit C	−0.300	−0.511
TF	0.983	0.182
TP	0.964	0.264
TT	1.000	0.014
FRAP	0.875	0.165
Rutin	0.564	0.652
p-coumaric	−0.715	0.457
Lightness (L*)	0.945	0.315
Chroma (C*)	−0.984	0.174
Hue angle (h°)	−0.791	0.303
Redness (a*)	−0.930	0.053
TCD	−0.551	0.821
MC_wb_	−0.969	0.236
RMC	−0.994	−0.114
Scores		
Freeze dried	6.147	0.816
40 °C	−1.067	−2.055
50 °C	−1.511	−1.356
60 °C	−3.569	2.595

*RSA* radical scavenging activity, *Vit C* Vitamin C, *TF* total flavonoid, *TP* total phenolic, *TT* total tannin, *FRAP* ferric reducing antioxidant power, *TCD* total color difference, *MC*_*wb*_ moisture content (wet basis), *RMC* residual moisture content

## Conclusions

The results of the study showed that drying processes have an impact on the bioactive compounds of pomegranate peel. Freeze drying peels had a positive effect on the total phenolic, tannins and flavonoid than oven drying at all temperature range. Moreover, freeze drying had a positive impact on the +catechin, -epicatechin, hesperidin and rutin concentrations of fruit peel. Pomegranate fruiot obtained from all the drying methods investigated were less effective against tyrosinase activity; however, they exhibited the best MIC against all the test bacteria. In addition, drying peels at 50 °C had a positive influence on the inhibitory activity of peel extracts against monophenolase. The results of the present study reveal that freeze-drying can be explored as a viable method for processing pomegranate peel to retain the maximum amount of their naturally occurring bioactive compounds.

### Abbreviations

%, percentage; °C, Degree celsius; CIE, Commission Internationale de l’Eclairage; L, CIE lightness coordinate; a*, CIE red(+)/green(−) colour attribute; b*, CIE yellow(+)/blue(−) colour attribute; C*, chroma; h°, hue angle; LC-MS, liquid chromatography–mass spectrometry; LC-MS^E^, liquid chromatography–mass spectrometry elctroscopy; TPC, total phenolic concentration; TFC, total flavonoid concentration; TTC, total tannins concentration; RSA, radical scavenging activity; FRAP, ferric reducing antioxidant power; DOPA, dihydroxyphenylalanine; mg CE/kg, milligram catechin equivalent per kilogram; mg/kg, milligram per kilogram; μg AAE/g, microgram ascorbic acid equivalent per gram; mg/ml, milligram per millilitre; POMOSA, Pomegranate Association of South African; mm, millimetre; AOAC, Association of Official Analytical Chemists; nr, number; m/s, meter per second; h, hour; ∆E*, total colour difference; ∆, change in measured attribute; g, gram; w/v, weight per volume; UPLC, ultra-performance liquid chromatograph; PDA, photo diode array; V, volt; kV, kilovolt; L/h, liter per hour; μm, micrometer; μl, microliter; ml/min, millimeter per minute; v/v, volume per volume; Folin-C, Folin-Ciocalteu; nm, nanometer; UV, ultraviolet; DM, dry weight; GAE, gallic acid equivalent; PVPP, polyvinylpolypyrrolidone; s, second; DPPH, 2, 2-diphenyl-1-picryl hydrazyl; mM, millimolar; μM, micromolar; pH, potential of hydrogen; TPTZ, 2,4,6-tripyridyl-s- triazine; FeCl_3_, iron(III) chloride; PJ, pomegranate juice; TE, trolox equivalent; MH, Mueller Hinton_; L_-DOPA, L-3,4-dihydroxyphenylalanine; DMSO, dimethyl sulfoxide; IC_50_, effective concentration at 50 %; EC50, effective concentration at 50 %; PCA, principal component analysis; MIC, minimum inhibitory concentration.
